# The Flipped Classroom Approach: A Feasible Way to Teach the Physical Exam in Spanish

**DOI:** 10.15766/mep_2374-8265.11532

**Published:** 2025-06-04

**Authors:** Andrew Bueno, Helen Woolcock Martinez, Ana I. Esteban-González

**Affiliations:** 1 First-Year Resident, Beth Israel Deaconess Medical Center; 2 Fourth-Year Medical Student, Columbia University Vagelos College of Physicians and Surgeons; 3 Assistant Professor of Community Health, Columbia University Irving Medical Center; † Co-primary author

**Keywords:** Language-Appropriate Health Care, Spanish, Physical Examination, Flipped Classroom, Communication Skills, Cultural Competence, Health Equity

## Abstract

**Introduction:**

There is an increasing need for medical Spanish education, and experts have suggested a flipped classroom approach incorporating online and in-person learning as a low-cost and effective way to teach language skills. As such, we produced a brief hybrid learning workshop to teach performance of the physical exam in Spanish.

**Methods:**

We developed a flipped classroom Spanish physical exam workshop that consisted of asynchronous online video modules and in-person teaching. The workshop was offered at a single medical school to all second-year preclinical students. Students were surveyed on their comfort performing a physical exam in Spanish (without an interpreter) before and after the in-person workshop.

**Results:**

A total of 62 students attended the workshop and completed surveys. Eighteen students (29%) watched some or all of the videos prior to the workshop and were highly satisfied with the quality of the videos (94% satisfied). After completing the in-person workshop, students reported significantly higher comfort in interacting with Spanish-speaking patients. Students reported increased comfort using an interpreter but also reported increased comfort with talking to patients without an interpreter present.

**Discussion:**

Overall, this intervention provides a feasible method for teaching the physical exam in Spanish and increasing students' comfort levels in working with Spanish-speaking patients. The Spanish proficiency levels for which this workshop might be most effective remain unclear. Care should be taken to ensure students are aware of their limitations and practice their language skills only in the presence of a certified medical interpreter.

## Educational Objectives

By the end of this activity, learners will be able to:

1. Introduce themselves and their role to patients in Spanish.
2. Use Spanish phrases to perform a thorough and efficient physical exam.
3. Recognize the importance of collaborating with a qualified interpreter.

## Introduction

For the past several decades, there has been an increase in the number of Spanish speakers in the United States. According to the US census, from 2000 to 2019 the number of individuals who speak Spanish at home increased by 57%.^1^ There has been a corresponding surge in demand for medical Spanish education as medical schools in the US search for ways to train students to effectively communicate with a disproportionately low-income and uninsured/underinsured Spanish-speaking population.^2^ This demand is reciprocated on the patients' side as well. In fact, approximately 80% of Spanish-speaking Hispanic adults in the US would prefer to see a Spanish-speaking physician.^3^

Much of the impetus for medical Spanish education comes from research showing the negative impact of language barriers on health care quality and improved outcomes with provider-patient concordance in language.^4,5^ For example, patients with limited English proficiency who have language-concordant providers have reported a better overall experience and satisfaction with care, increased medication compliance, and improved understanding of diagnoses.^6–9^ Spanish-speaking patients' issues and concerns are more likely to be elicited by physicians who are fluent in Spanish, compared to less fluent counterparts or colleagues who used professional interpreters.^10^

Given these benefits and the fact that the most common second language in many medical centers in the US is Spanish, more medical schools have begun to incorporate some form of medical Spanish into their curriculum. In one study from 2021, of the 125 of 158 medical schools that responded to study surveys, 78% offered medical Spanish programming to some extent, with 54% having formal curricula.^11^ Despite this encouraging trend, barriers persist that prevent students from getting appropriate and high-quality medical Spanish education.^11^ Schools have cited difficulty with finding qualified faculty and time in the curriculum to teach medical Spanish.^12^ Some have also expressed concerns about the risk of false fluency in settings where students may overestimate their skills in working with Spanish-speaking patients, leading to patient safety issues.^12^

Due to these barriers, as well as the difficulties of the COVID-19 pandemic, scholars in recent years have begun to explore online options for medical Spanish education, emphasizing the benefits of democratizing information to reach a wider audience while working with limited resources.^13^ Many are enthusiastic about the flipped classroom approach, in which online modules are combined with in-person interactive workshops to solidify learning and give students a chance to receive real-time feedback.^13^

In thinking about which aspects of medical Spanish education are highest yield for teaching effective patient communication skills and most conducive to the flipped classroom approach, the physical exam stands out for several reasons. For one, in our experience, the number of actual phrases and vocabulary that one needs to use while conducting a physical exam is relatively small, whereas visual aids, such as demonstrating the maneuvers, can be useful in explaining the exam, and many of the same phrases can be generalized to other parts of the physical exam.^14,15^ In addition, physical exams conducted in a patient's language can be markedly more efficient and seamless than performing an exam with the assistance of a phone interpreter who cannot see the patient.^16^ Within the constraints of clinical care, compared to use of an interpreter, the time saved by giving directions to patients in Spanish can be dedicated to eliciting a more thorough history review and physical exam.^16^

While we have identified some online demonstrations of the physical exam in Spanish,^17^ we have yet to find a comprehensive set of videos that has been assessed for effectiveness and acceptability. We aimed to evaluate whether a flipped classroom approach to the physical exam is feasible, and whether such an approach would be well received by preclinical medical students and would allow students to feel better prepared to work with Spanish-speaking patients, with the help of an interpreter, during their clinical year.

## Methods

We implemented the modules and workshop at the Vagelos College of Physicians and Surgeons (VP&S) at Columbia University, a private medical school situated in the Washington Heights/Inwood neighborhood of northern Manhattan, where Spanish is the primary language of 62% of households.^18^

The module videos and in-person workshop were developed by two medical students with certified fluency in medical Spanish (Andrew Nicholas Bueno, Helen Woolcock Martinez) through the LanguageLine electronic Bilingual Fluency Assessment. The workshop was overseen by a faculty member (Ana I. Esteban-González) who completed her medical training in Spain and who has several decades of experience in medical Spanish education. The content of the videos was based on the Dígame Más Curriculum developed by the faculty supervisor (Ana I. Esteban-González), drawing from materials developed in-house and revised over years of teaching Spanish by body system to medical students at Columbia University.^19^ The videos themselves were recorded in the VP&S simulation center.

We included the workshop within the Ready for Major Clinical Year curriculum, a preclinical second-year longitudinal curriculum that prepares students for their clinical year through simulation sessions and lectures on topics ranging from appropriate interpreter use to writing hospital notes. While this is a required curriculum for all students, attendance was not taken, per course policy. We emailed students to inform them of the in-person workshop and included links to the module videos (Appendices A–G) as preliminary work to gain exposure to the Spanish vocabulary that would be reinforced throughout the workshop. These module videos were highly encouraged but not mandatory. All second-year medical students (*N* = 140) of all Spanish fluency levels were encouraged to attend.

The physical exam workshop was an in-person event conducted in a lecture hall capable of seating approximately 250 people. Students were not asked or required to bring physical exam tools (stethoscope, tuning fork, etc.), as this workshop was focused more on verbal Spanish skills rather than examination skills. The physical exam workshop consisted of a brief overview, followed by two demonstrations of a comprehensive head-to-toe physical exam in Spanish on an instructor serving as a practice patient. In the first demonstration, the session leader explained each step of the exam. Students were able to ask questions throughout. The second demonstration was a fluid run-through of the entire exam contextualized with a case-based scenario. In the latter half of the workshop, students were given time to practice performing a physical exam on each other, while five student volunteers, also certified in medical Spanish through the electronic Bilingual Fluency Assessment test, and the two student leaders (Andrew Nicholas Bueno and Helen Woolcock Martinez) circulated around the room to answer questions and provide feedback. More detailed information on workshop characteristics and design can be found in the Figure.

**Figure.**
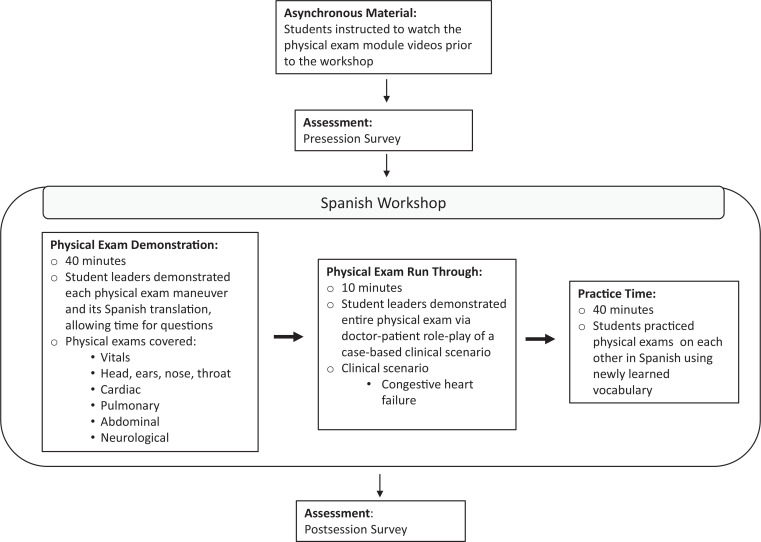
Design and workflow of the medical Spanish physical exam workshop, utilizing a flipped classroom curriculum structure.

Throughout the workshop, students were reminded that they should always have an interpreter on the line, unless they have been certified to speak Spanish with patients. Students had previously received a lecture on appropriate interpreter use, which also delineated patient Title VI civil rights to language-concordant medical care, the importance of interpreter use for patient outcomes, and techniques for recognizing one's limitations when speaking Spanish. This lecture was already included in the Ready for Major Clinical Year curriculum prior to the introduction of the physical exam workshop.

Students were given a short handout outlining each part of the physical exam interaction, which included high-yield Spanish phrases to use for reference (Appendix H). In terms of other materials/equipment, we used a projector for the slide deck (Appendix I) and microphones for the instructor and the practice patient.

To assess the student-perceived impact of this medical Spanish education workshop and modules, we designed pre- and postworkshop surveys (Appendix J). The surveys assessed student attitudes toward medical Spanish learning (preworkshop; rated on 5-point Likert scale [1 = *strongly disagree*, 5 = *strongly agree*]), satisfaction with the preworkshop video modules (preworkshop; rated on 5-point Likert scale [1 = *very unsatisfied*, 5 = *very satisfied*]), comfort in clinical scenarios with Spanish-speaking patients (pre- and postworkshop; rated on 5-point Likert scale [1 = *very uncomfortable*, 5 = *very comfortable*]), and satisfaction with the workshop (postworkshop; rated on 5-point Likert scale [1 = *very unsatisfied*, 5 = *very satisfied*]). In addition, the surveys included two open-ended questions to elicit feedback.

These anonymous surveys were granted exemption by the institutional review board at Columbia University and were approved for administration to students by the dean of education of the medical school.

We employed descriptive statistics to outline parameters of the sample/responses. Paired *t* tests were used to compare pre- and postworksop Likert-scale survey response variables.

## Results

Of the 140 second-year medical students at Columbia University, 44% (*n* = 62) both (1) attended the workshop and (2) filled out the pre- and postworkshop surveys. Of those who filled out the surveys, 29% (*n* = 18) reported watching the videos prior to attending the workshop.

### Student Attitudes

Prior to the workshop, 89% of students (*n* = 55) agreed that learning Spanish is important to them, and 92% (*n* = 57) agreed that they would like to learn Spanish to better communicate with their future patients. Additionally, 90% (*n* = 56) agreed that more physicians in the US should learn how to speak Spanish.

### Physical Exam Module Videos

Of the 18 students that reported watching the preworkshop module videos, 83% (*n* = 15) were satisfied with the difficulty and usefulness of the phrases used in the videos, and 94% (*n* = 17) were satisfied with the quality of the videos (Table 1).

**Table 1.**
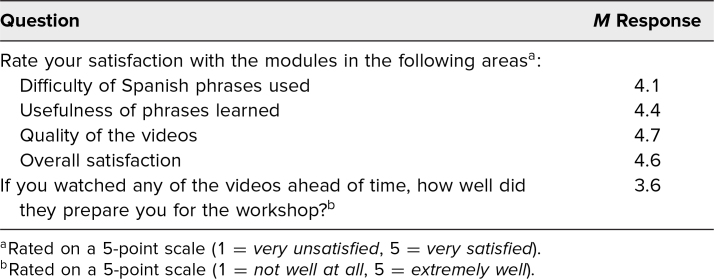
Student Satisfaction With the Module Videos and Preparedness For the Spanish Physical Exam Workshop (*n* = 18)

### Workshop Student Impact

Data on the workshop's impact on comfort levels can be found in Table 2. We found that after participating in the physical exam workshop, students felt more comfortable introducing themselves and building rapport in Spanish. Student comfort levels with performing a thorough and efficient physical exam in Spanish increased from pre- to postworkshop, with comfort scores increasing by a mean 1.1 points on a 5-point Likert scale. While students felt more comfortable collaborating with an interpreter after the workshop, they also reported greater comfort in talking with patients without an interpreter present, which was not one of the goals of the workshop. Overall, this set of questions had a Cronbach's alpha of .854, indicating a high level of internal consistency.

**Table 2.**
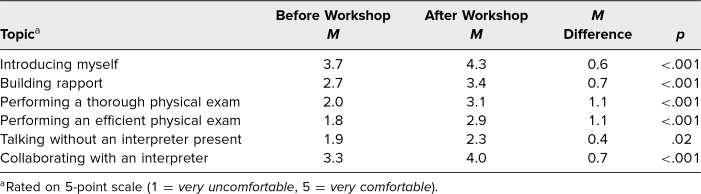
Comparison of Student Pre- and Postworkshop Comfort With Various Aspects of the Patient Physical Exam in Spanish (*N* = 62)

## Discussion

We designed and executed a brief hybrid Spanish physical exam workshop, with the evaluation of our survey results indicating that this is a feasible and acceptable method for increasing students' comfort levels in using basic Spanish physical exam phrases.

Recent research has highlighted the importance of medical Spanish curricula to enhance students' comfort level and language skills in working with Spanish-speaking patients. For the most part, these studies have focused on longitudinal curricula, with courses lasting anywhere from 4 weeks up to 18 months, finding that students' self-reported comfort levels were much greater once students had participated in a rigorous medical Spanish curriculum.^20–22^ Other medical Spanish programs have demonstrated that students' self-confidence in utilizing Spanish skills increased after only 8 hours of engagement with a topic-focused educational module.^23,24^ Our results show that this workshop significantly increased student-reported levels of comfort with engaging with Spanish-speaking patients over a much shorter time course. Students began the workshop feeling generally *somewhat uncomfortable* and ended the workshop feeling generally *neutral* about being able to perform a thorough physical exam in Spanish. These findings are to be expected, given that we provided students with the tools to execute a basic physical exam rather than a comprehensive physical exam. Aside from an expected increase in comfort with performing a physical exam in Spanish, students reported significantly higher confidence in building rapport with Spanish-speaking patients and introducing themselves in Spanish, skills that students can use to provide better care across many patient encounters and situations. This aligns with studies demonstrating that the benefits of using language-concordant health care providers include enhanced rapport building and increased patient satisfaction with their care.^25,26^ In this case, our students' higher comfort in building rapport likely comes from feeling more comfortable using high-yield phrases for effective communication, such as introducing oneself and giving the patient context for the clinical encounter (i.e., explaining that they will perform a physical exam).

In acknowledging the power of this intervention to increase student comfort in working with Spanish-speaking patients, it is extremely important to outline the steps that we have taken to ensure patient safety in the development process; namely, we acknowledge the hazard of false fluency, wherein a student overestimates their skills in working with Spanish-speaking patients, leading to miscommunication.^27^ This was of particular concern when developing this workshop, as the shorter 120-minute time frame allows for less time for students to practice and master the vocabulary presented. As such, in creating the modules, we addressed this concern in several ways. First, we ensured that the workshop took place within a curriculum in which students learned the technique and importance of proper interpreter use, including the importance of recognizing one's own limitations (this was addressed in a previous lecture that students received; the materials are not included in this publication). Second, we emphasized multiple times during the workshop itself that one must always have an interpreter on the line when speaking Spanish with a patient, unless they have been specifically and formally certified to speak Spanish in a medical context. Lastly, and relatedly, the workshop was conducted in the context of a medical school and hospital system that requires formal certification for students to be able to speak with patients without an interpreter.

Assessment of students' language competency is an important and often forgotten aspect of medical Spanish language education that is critical to augment students' learning and prevent false fluency.^12^ Before and after the workshop, students were reminded of this competency requirement, and those interested in receiving Spanish language certification were given the opportunity to start the process. Concerningly, we did see a small but statistically significant increase from before to after the workshop (mean 0.4-point increase on 5-point Likert scale; *p* = .02) in student comfort with speaking with a patient without an interpreter present. Although it is the smallest effect size that we observed among all of the questions pertaining to comfort levels, it is important to ask whether there is more that needs to be done to clarify for students the importance of interpreter use and the inappropriateness of talking without a certified interpreter present. One possible reason for the increase in this metric was that some students may have interpreted the question as referring to nonmedical small talk rather than information of medical importance. In future workshops, the survey will be clarified to assess specifically talking to patients about medical information without an interpreter present. We plan to continue to include this question to monitor the workshop's effectiveness at preventing false fluency. We also strongly recommend and urge those who have watched the videos and/or attended the workshop to continue using interpreters in all clinical spaces.

Aside from the workshop itself, we also aimed to assess the feasibility, quality, and efficacy of the asynchronous online modules we developed to supplement the in-person learning. It was important for us to evaluate the flipped classroom approach of hybrid learning, which aligned with expert recommendations on effective online medical Spanish resources.^28^ Our survey data demonstrated high levels of satisfaction with the online modules for students who viewed them before the workshop. Our findings support the claim that online modules are a well-received, low-cost way to expand access to medical Spanish education for students, thus improving the health care experience for Spanish-speaking patients.^20^ Contextualizing the modules with the in-person workshop allowed students to apply and solidify their learning while receiving real-time feedback on their skills.

To our knowledge, our modules and workshop are the only resource in the medical education literature to be designed and evaluated by medical students for teaching the physical exam in Spanish. Its strengths include faculty and student instructors who were certified to be competent in medical Spanish, an important aspect of effective language education.^12^ Once developed, the online modules and workshop were low-cost and low-time intensive interventions to implement. Aside from space to perform the workshop and several volunteers to help facilitate, the only other material cost was printed handouts, which in the future could be easily replaced by an online PDF form if all students are able to bring an internet-capable device.

Our workshop design also assessed student attitudes toward medical Spanish education prior to the workshop. This assessment is important to understanding students' baseline motivations and biases regarding Spanish language learning, which may impact their perception of the acceptability and efficacy of the modules and workshop. Future workshops may consider analyzing workshop survey data based on students' ratings of the importance of learning Spanish.

Finally, our modules and workshops represent a critical advancement in integrating Spanish language education into medical school curricula, addressing the need for culturally and linguistically competent health care providers in the US.^29^

There are several aspects of the study that may limit its interpretation. Our participants represented a narrow group of learners, specifically preclinical medical students about to start clinical rotations who were knowledgeable about the physical exam and who, for the most part, highly believed in the importance of medical Spanish education. Caution should be applied in expecting similar results for students who are less motivated or are in other stages of their training. In terms of our survey data, some of the survey questions were vague in nature, such as asking students to rate the quality of the videos or the usefulness of the phrases learned. Future iterations of these workshops may consider asking more directed survey questions that elicit the precise level of content acquisition and student-rated confidence levels in applying what they learned in the workshop to the clinical space.

Another limitation is that our survey did not differentiate participants based on preexisting fluency; thus, it is unclear whether the effects of the workshop were shared equally across fluency levels. Understanding participants' self-reported preworkshop fluency levels would have also allowed us to better tailor the workshop around student proficiency levels and analyze our results to elucidate which proficiency levels may be most useful for student participation in this workshop. This limitation notwithstanding, students of all proficiency levels were deliberately included in the workshop, with the idea that by practicing in a group with many different Spanish competency levels, everyone benefits. Future iterations of this workshop should include survey questions that assess students' self-reported proficiency levels to determine the effects of the intervention for beginning, intermediate, and advanced learners. Relatedly, another aspect of this study that may limit its validity in other student groups is that the students were already educated on appropriate interpreter use, both for in-person and phone interpreters, as a part of their preclinical curriculum training. However, this may not be the case for other students at other institutions that may be interested in implementing these workshops. As such, along with emphasizing the importance of interpreter use throughout the workshop, it is also necessary that students receive a lecture on how to appropriately use interpreters in clinical scenarios and how interpreter use impacts patient outcomes.

Lastly, interpretations may be limited based on student attendance and engagement with the materials within the study design. Survey data were able to capture feedback from almost half of the entire second-year preclinical class, but the data did not include students who did not come to the session (school policy did not require that attendance be taken) and did not include students who attended but did not fill out the surveys. In a similar vein, the percentage of students who viewed the online modules beforehand was relatively low (29%). Students were highly encouraged but not required to watch the videos (total length of videos was approximately 40 minutes). In the future, these modules should be mandatory and protected time should be allotted in students' schedules to view the modules, which is in line with prior research showing that this strategy is associated with better completion rates.^30^ The students who did choose to watch the videos beforehand were satisfied with the quality, which is encouraging that students may stand to benefit even more once these measures are taken in the future to increase student engagement with the asynchronous portion.

## Appendices


Introduction.mp4Vitals.mp4Cardiovascular.mp4Pulmonary.mp4Abdominal.mp4HEENT.mp4Neuro.mp4Workshop Student Handout.pptxWorkshop Slideshow.pptxSession Surveys.docx
*All appendices are peer reviewed as integral parts of the Original Publication.*
