# Supplementary material for: The Flipped Classroom Approach: A Feasible Way to Teach the Physical Exam in Spanish
Source: MedEdPORTAL. 2025 Jun 4;21:11532. doi: 10.15766/mep_2374-8265.11532 (PMC12134118; doi:10.15766/mep_2374-8265.11532)
Supplement: Supplementary file 1 — Introduction.mp4Vitals.mp4Cardiovascular.mp4Pulmonary.mp4Abdominal.mp4HEENT.mp4Neuro.mp4Workshop Student Handout.pptxWorkshop Slideshow.pptxSession Surveys.docx [file mep_2374-8265.11532-s001.zip › H. Workshop Student Handout.pptx]

## Slide 1
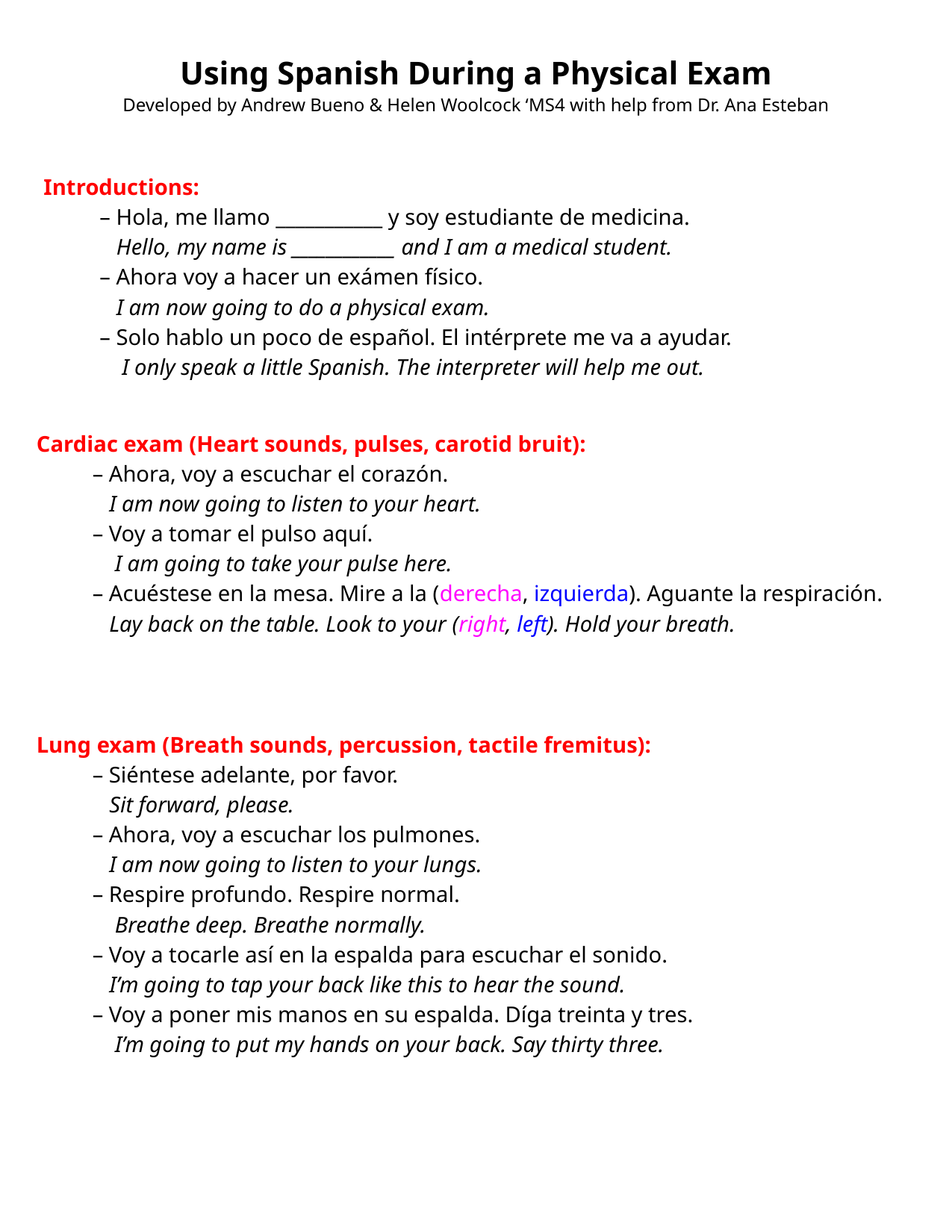

# Using Spanish During a Physical Exam
Developed by Andrew Bueno & Helen Woolcock ‘MS4 with help from Dr. Ana Esteban
Introductions:
– Hola, me llamo ___________ y soy estudiante de medicina.
 Hello, my name is ____________ and I am a medical student.
– Ahora voy a hacer un exámen físico.
 I am now going to do a physical exam.
– Solo hablo un poco de español. El intérprete me va a ayudar.
 I only speak a little Spanish. The interpreter will help me out.
Cardiac exam (Heart sounds, pulses, carotid bruit):
– Ahora, voy a escuchar el corazón.
 I am now going to listen to your heart.
– Voy a tomar el pulso aquí.
 I am going to take your pulse here.
– Acuéstese en la mesa. Mire a la (derecha, izquierda). Aguante la respiración.
 Lay back on the table. Look to your (right, left). Hold your breath.
Lung exam (Breath sounds, percussion, tactile fremitus):
– Siéntese adelante, por favor.
 Sit forward, please.
– Ahora, voy a escuchar los pulmones.
 I am now going to listen to your lungs.
– Respire profundo. Respire normal.
 Breathe deep. Breathe normally.
– Voy a tocarle así en la espalda para escuchar el sonido.
 I’m going to tap your back like this to hear the sound.
– Voy a poner mis manos en su espalda. Díga treinta y tres.
 I’m going to put my hands on your back. Say thirty three.

## Slide 2
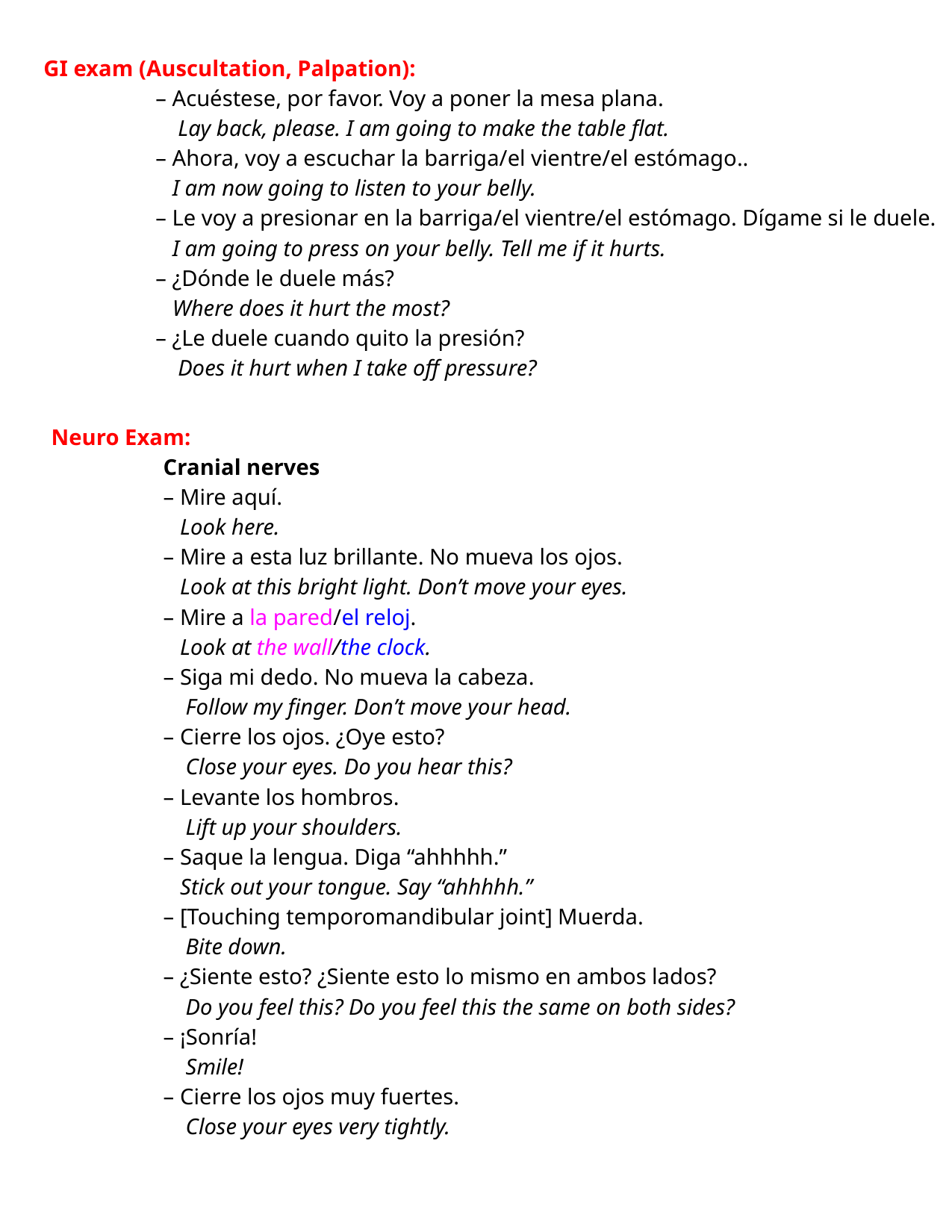

GI exam (Auscultation, Palpation):
	– Acuéstese, por favor. Voy a poner la mesa plana.
	 Lay back, please. I am going to make the table flat.
	– Ahora, voy a escuchar la barriga/el vientre/el estómago..
	 I am now going to listen to your belly.
	– Le voy a presionar en la barriga/el vientre/el estómago. Dígame si le duele.
	 I am going to press on your belly. Tell me if it hurts.
	– ¿Dónde le duele más?
	 Where does it hurt the most?
	– ¿Le duele cuando quito la presión?
	 Does it hurt when I take off pressure?
Neuro Exam:
	Cranial nerves
	– Mire aquí.
	 Look here.
	– Mire a esta luz brillante. No mueva los ojos.
 	 Look at this bright light. Don’t move your eyes.
	– Mire a la pared/el reloj.
	 Look at the wall/the clock.
	– Siga mi dedo. No mueva la cabeza.
	 Follow my finger. Don’t move your head.
	– Cierre los ojos. ¿Oye esto?
	 Close your eyes. Do you hear this?
	– Levante los hombros.
	 Lift up your shoulders.
	– Saque la lengua. Diga “ahhhhh.”
	 Stick out your tongue. Say “ahhhhh.”
	– [Touching temporomandibular joint] Muerda.
	 Bite down.
	– ¿Siente esto? ¿Siente esto lo mismo en ambos lados?
	 Do you feel this? Do you feel this the same on both sides?
	– ¡Sonría!
	 Smile!
	– Cierre los ojos muy fuertes.
 Close your eyes very tightly.

## Slide 3
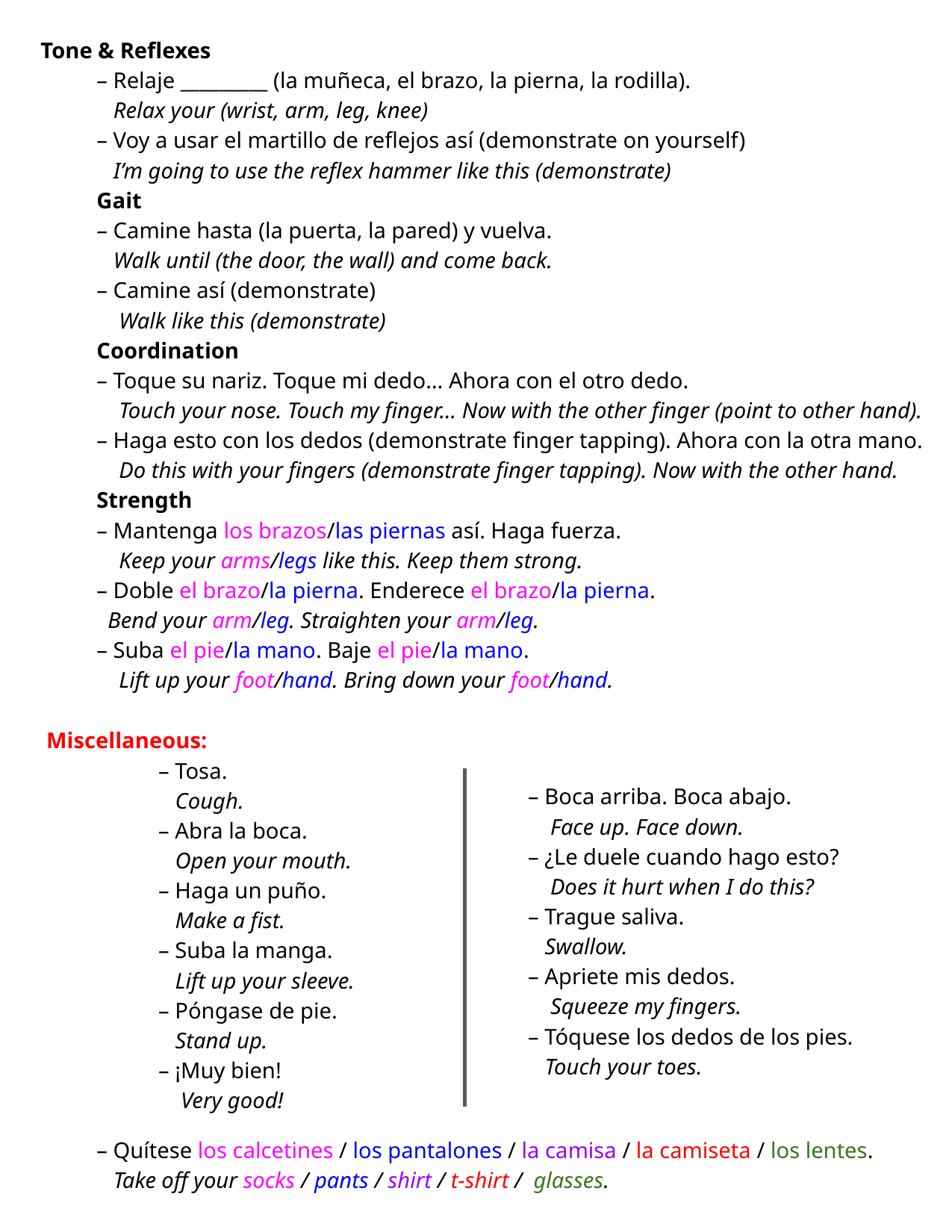

Tone & Reflexes
	– Relaje _________ (la muñeca, el brazo, la pierna, la rodilla). 	 	 	 Relax your (wrist, arm, leg, knee)
	– Voy a usar el martillo de reflejos así (demonstrate on yourself)
	 I’m going to use the reflex hammer like this (demonstrate)
	Gait
	– Camine hasta (la puerta, la pared) y vuelva.
	 Walk until (the door, the wall) and come back.
	– Camine así (demonstrate)
	 Walk like this (demonstrate)
	Coordination
	– Toque su nariz. Toque mi dedo… Ahora con el otro dedo.
	 Touch your nose. Touch my finger… Now with the other finger (point to other hand).
	– Haga esto con los dedos (demonstrate finger tapping). Ahora con la otra mano.
	 Do this with your fingers (demonstrate finger tapping). Now with the other hand.
	Strength
	– Mantenga los brazos/las piernas así. Haga fuerza.
	 Keep your arms/legs like this. Keep them strong.
	– Doble el brazo/la pierna. Enderece el brazo/la pierna.
 	 Bend your arm/leg. Straighten your arm/leg.
	– Suba el pie/la mano. Baje el pie/la mano.
	 Lift up your foot/hand. Bring down your foot/hand.
Miscellaneous:
	– Tosa.
	 Cough.
	– Abra la boca.
	 Open your mouth.
	– Haga un puño.
	 Make a fist.
	– Suba la manga.
	 Lift up your sleeve.
	– Póngase de pie.
	 Stand up.
	– ¡Muy bien!
	 Very good!
	– Boca arriba. Boca abajo.
	 Face up. Face down.
	– ¿Le duele cuando hago esto?
	 Does it hurt when I do this?
	– Trague saliva.
	 Swallow.
	– Apriete mis dedos.
	 Squeeze my fingers.
	– Tóquese los dedos de los pies.
	 Touch your toes.
	– Quítese los calcetines / los pantalones / la camisa / la camiseta / los lentes.
	 Take off your socks / pants / shirt / t-shirt / glasses.
