# Supplementary material for: The Flipped Classroom Approach: A Feasible Way to Teach the Physical Exam in Spanish
Source: MedEdPORTAL. 2025 Jun 4;21:11532. doi: 10.15766/mep_2374-8265.11532 (PMC12134118; doi:10.15766/mep_2374-8265.11532)
Supplement: Supplementary file 1 — Introduction.mp4Vitals.mp4Cardiovascular.mp4Pulmonary.mp4Abdominal.mp4HEENT.mp4Neuro.mp4Workshop Student Handout.pptxWorkshop Slideshow.pptxSession Surveys.docx [file mep_2374-8265.11532-s001.zip › I. Workshop Slideshow.pptx]

## Slide 1
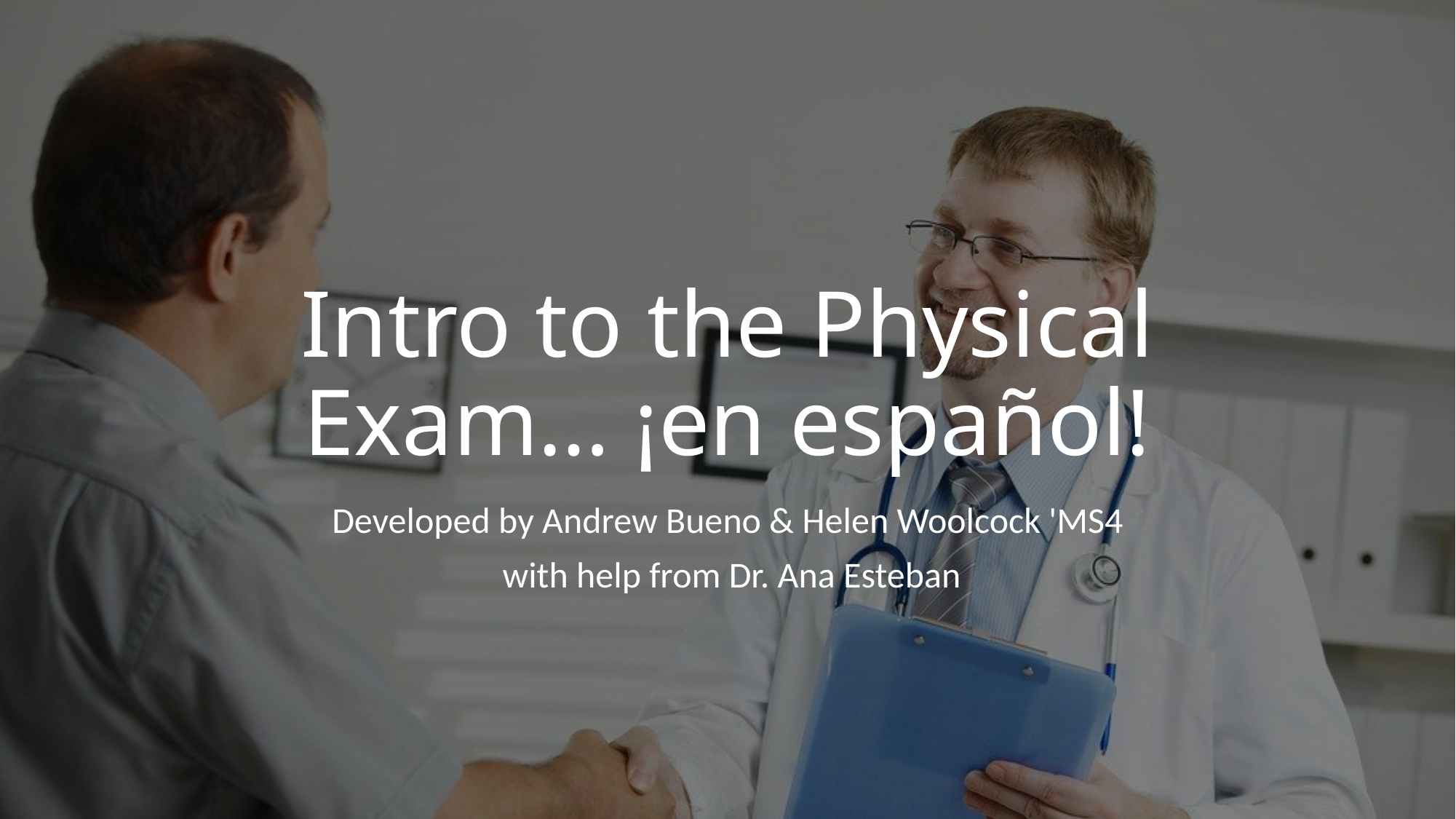

# Intro to the Physical Exam… ¡en español!
Developed by Andrew Bueno & Helen Woolcock 'MS4
 with help from Dr. Ana Esteban

## Slide 2
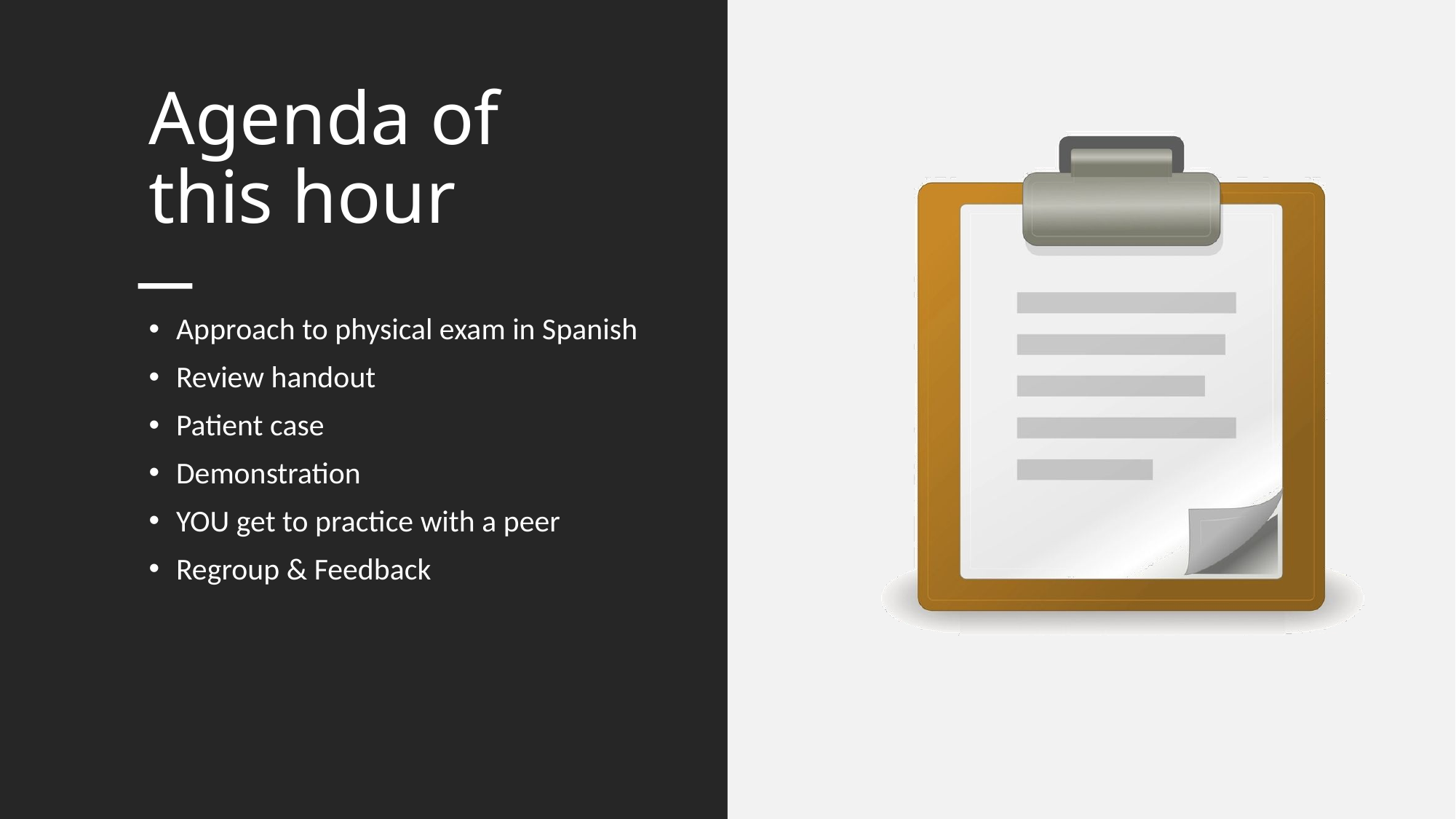

# Agenda of this hour
Approach to physical exam in Spanish
Review handout
Patient case
Demonstration
YOU get to practice with a peer
Regroup & Feedback

## Slide 3
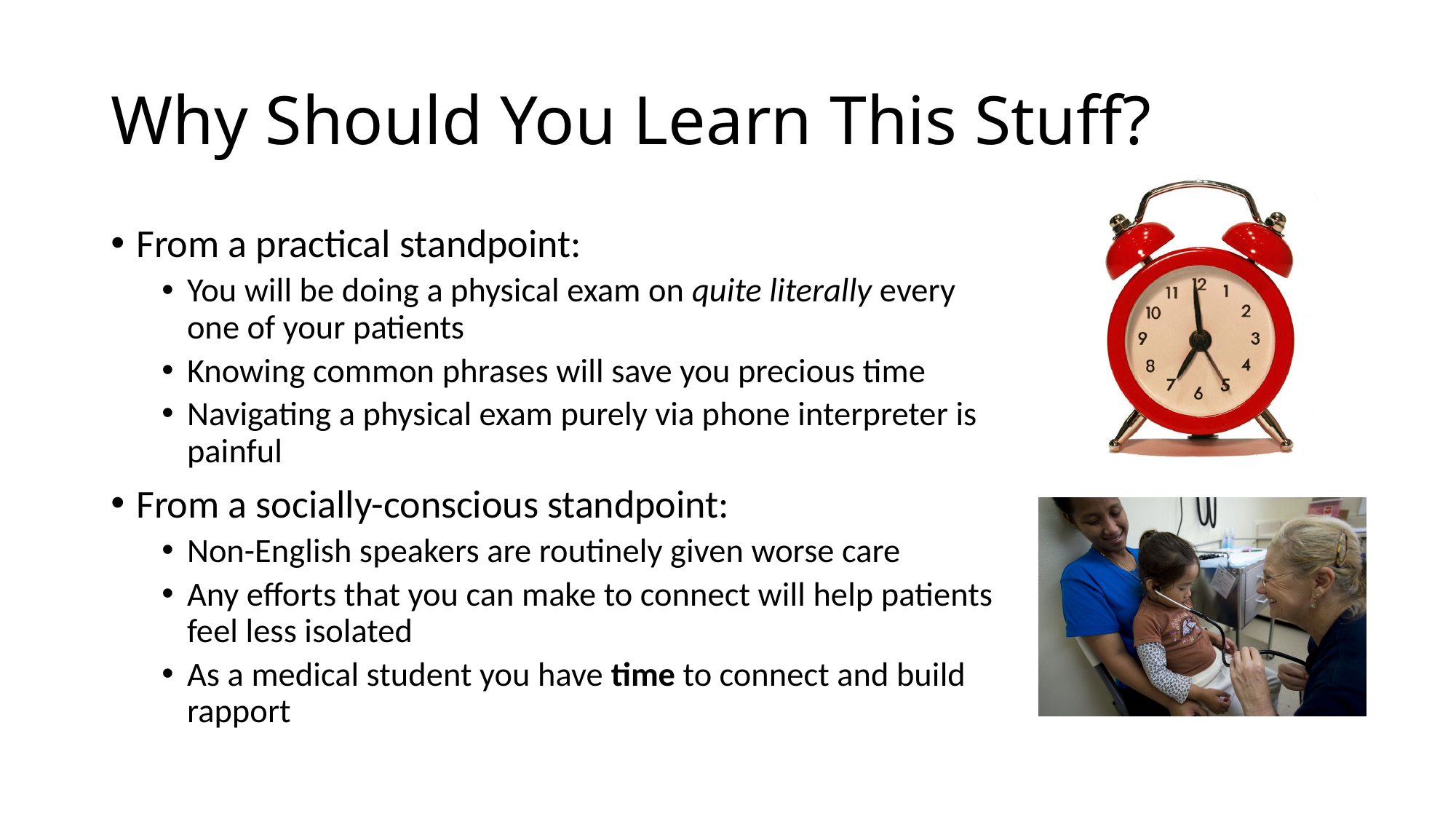

# Why Should You Learn This Stuff?
From a practical standpoint:
You will be doing a physical exam on quite literally every one of your patients
Knowing common phrases will save you precious time
Navigating a physical exam purely via phone interpreter is painful
From a socially-conscious standpoint:
Non-English speakers are routinely given worse care
Any efforts that you can make to connect will help patients feel less isolated
As a medical student you have time to connect and build rapport

## Slide 4
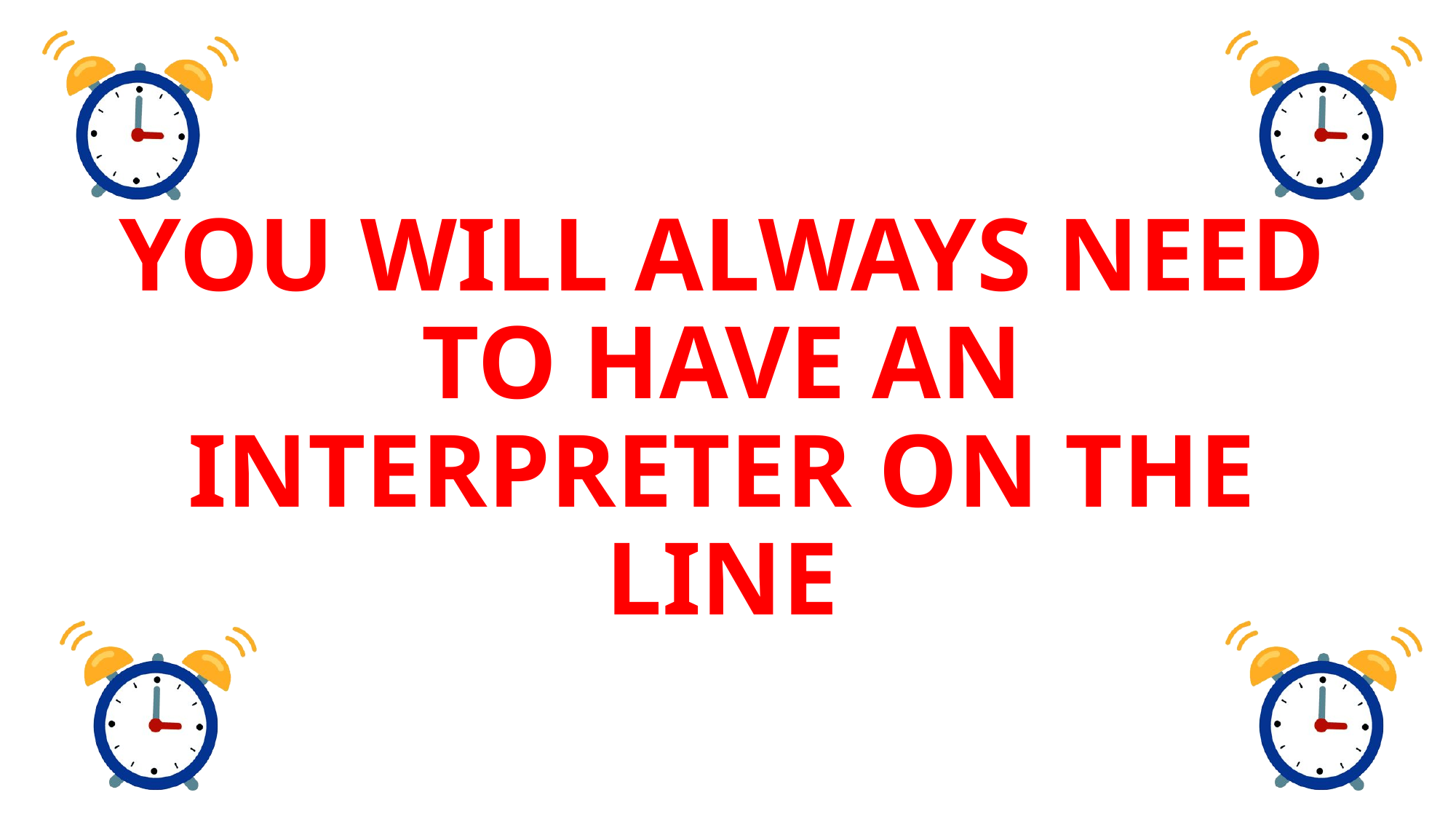

# YOU WILL ALWAYS NEED TO HAVE AN INTERPRETER ON THE LINE

## Slide 5
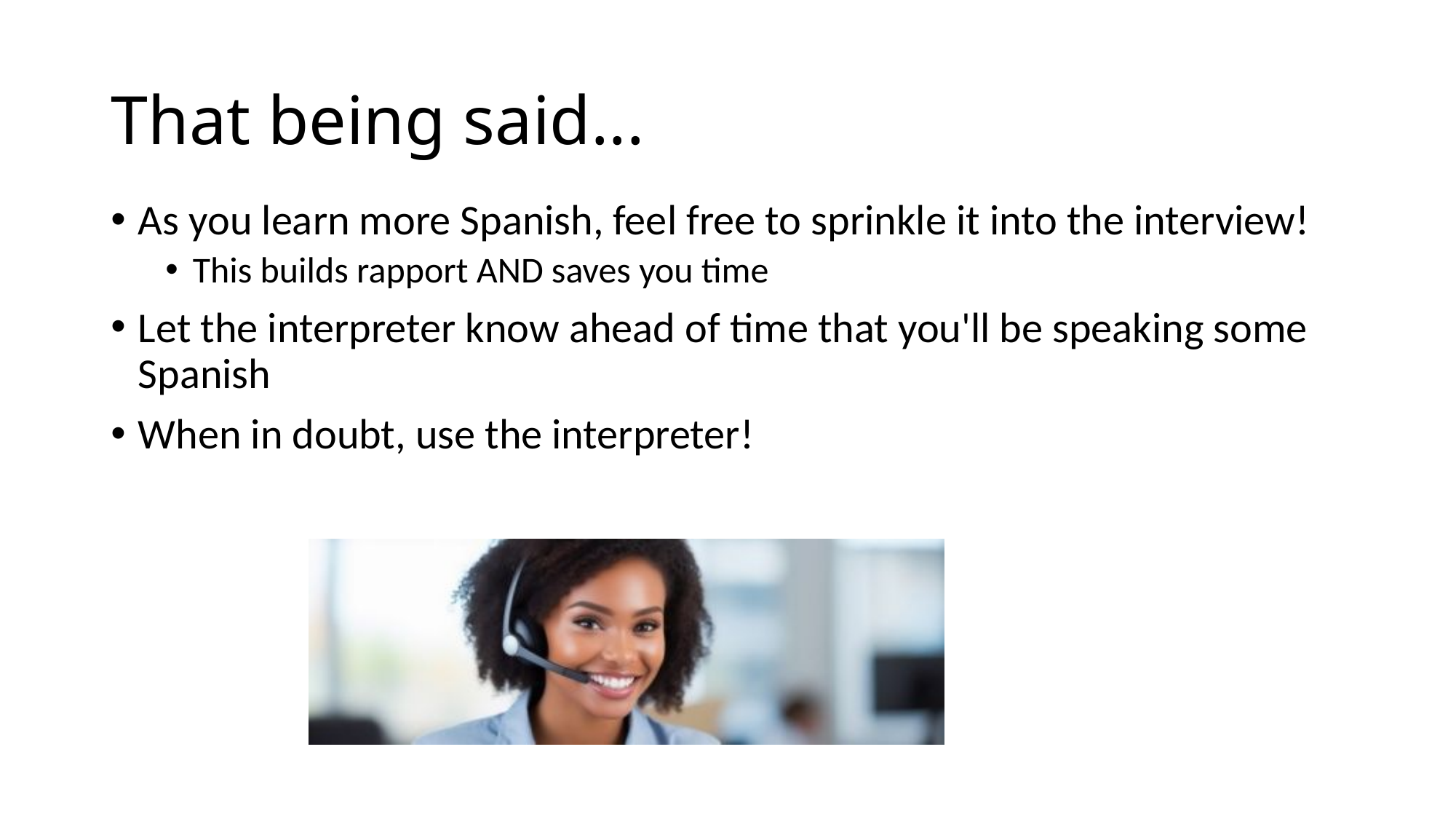

# That being said...
As you learn more Spanish, feel free to sprinkle it into the interview!
This builds rapport AND saves you time
Let the interpreter know ahead of time that you'll be speaking some Spanish
When in doubt, use the interpreter!

## Slide 6
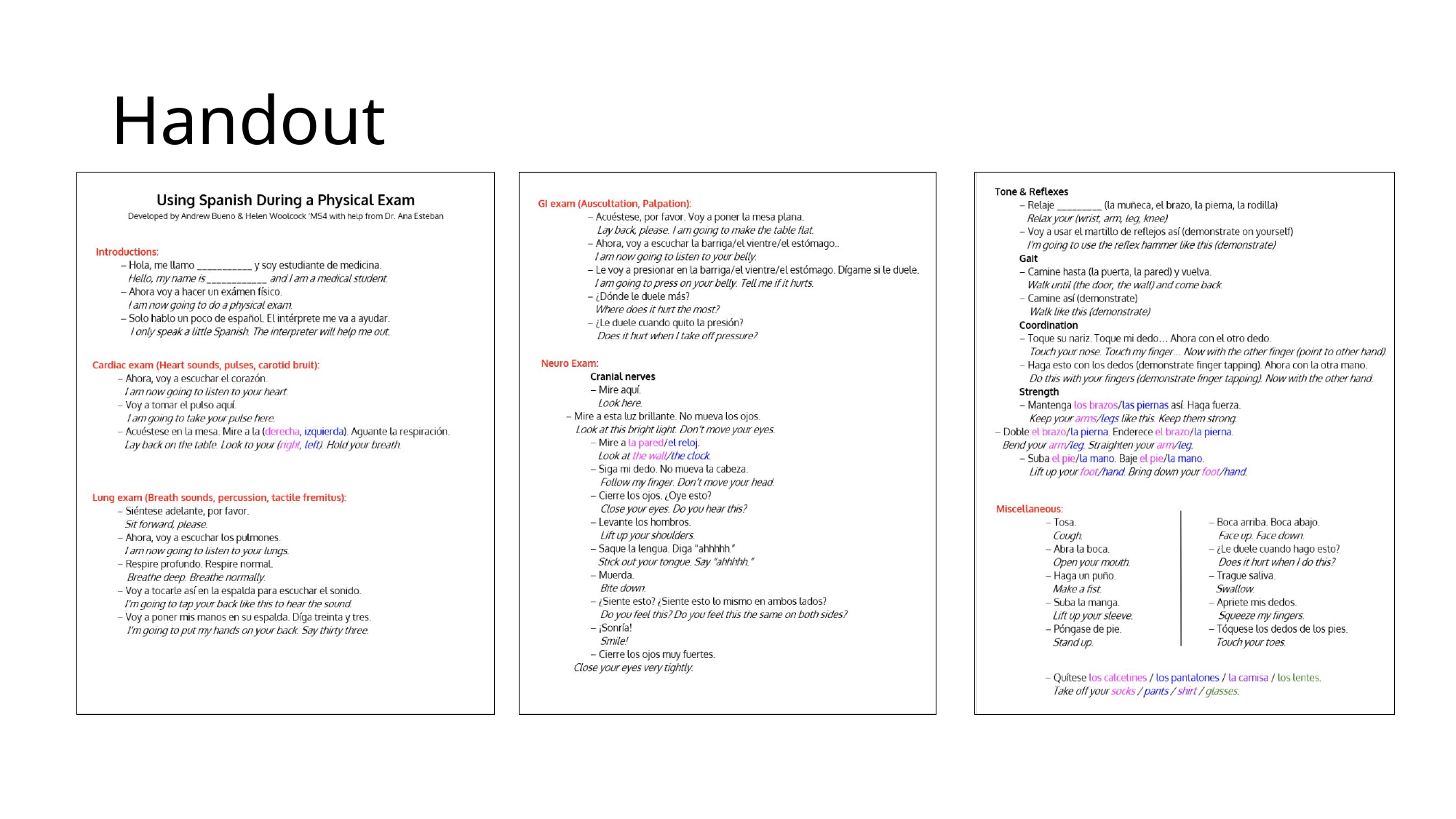

# Handout

## Slide 7
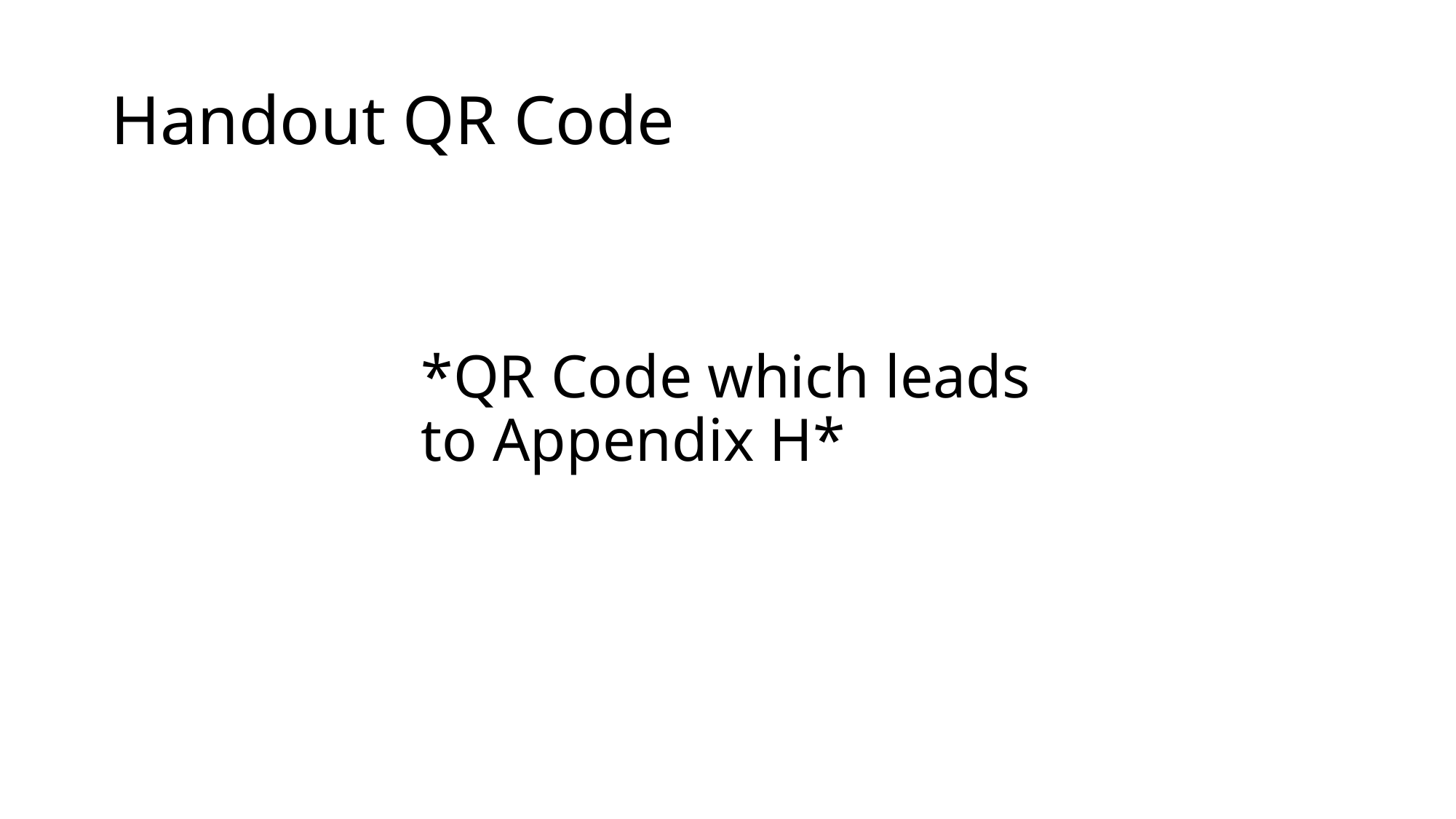

# Handout QR Code
*QR Code which leads to Appendix H*

## Slide 8
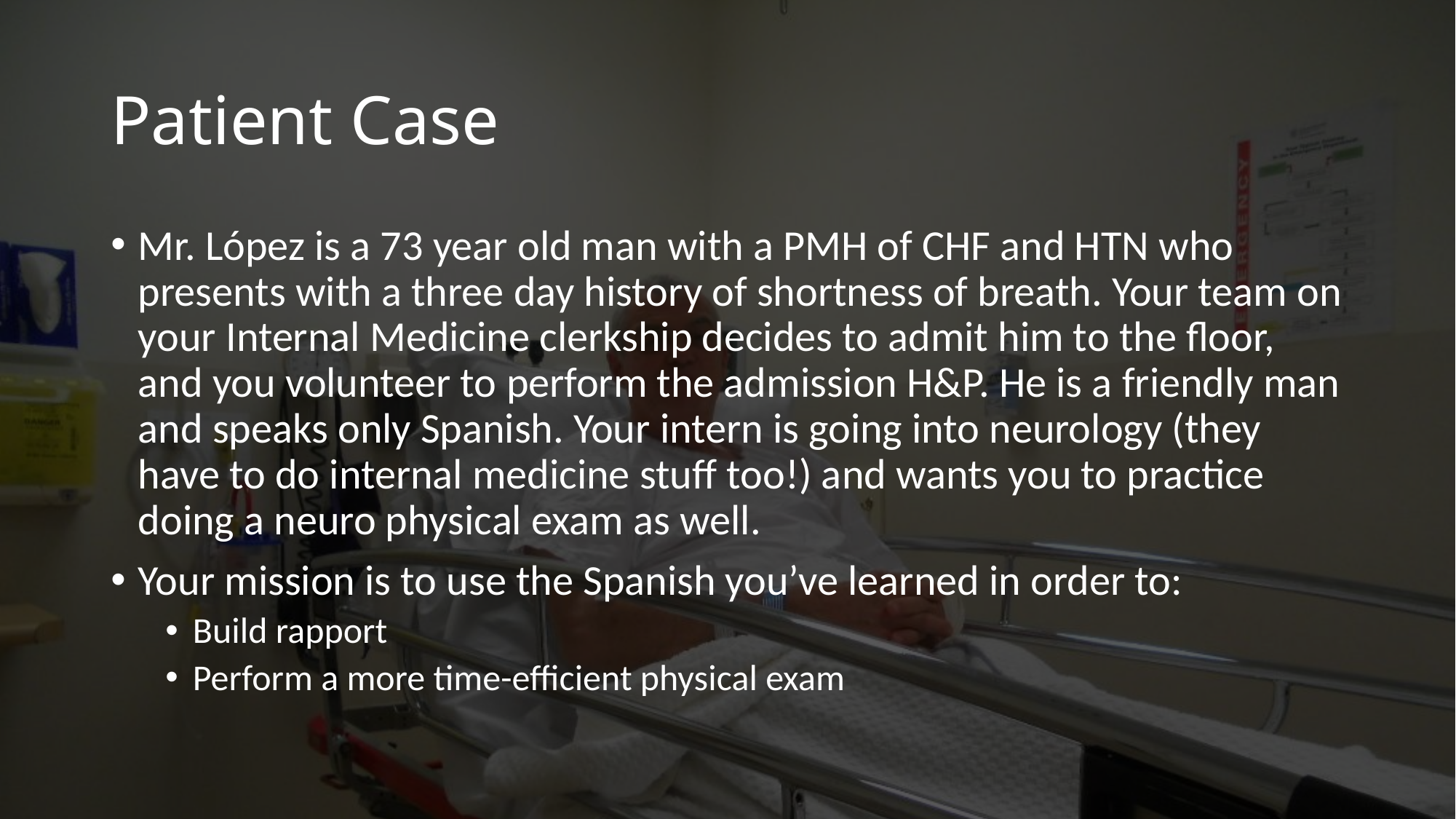

# Patient Case
Mr. López is a 73 year old man with a PMH of CHF and HTN who presents with a three day history of shortness of breath. Your team on your Internal Medicine clerkship decides to admit him to the floor, and you volunteer to perform the admission H&P. He is a friendly man and speaks only Spanish. Your intern is going into neurology (they have to do internal medicine stuff too!) and wants you to practice doing a neuro physical exam as well.
Your mission is to use the Spanish you’ve learned in order to:
Build rapport
Perform a more time-efficient physical exam

## Slide 9
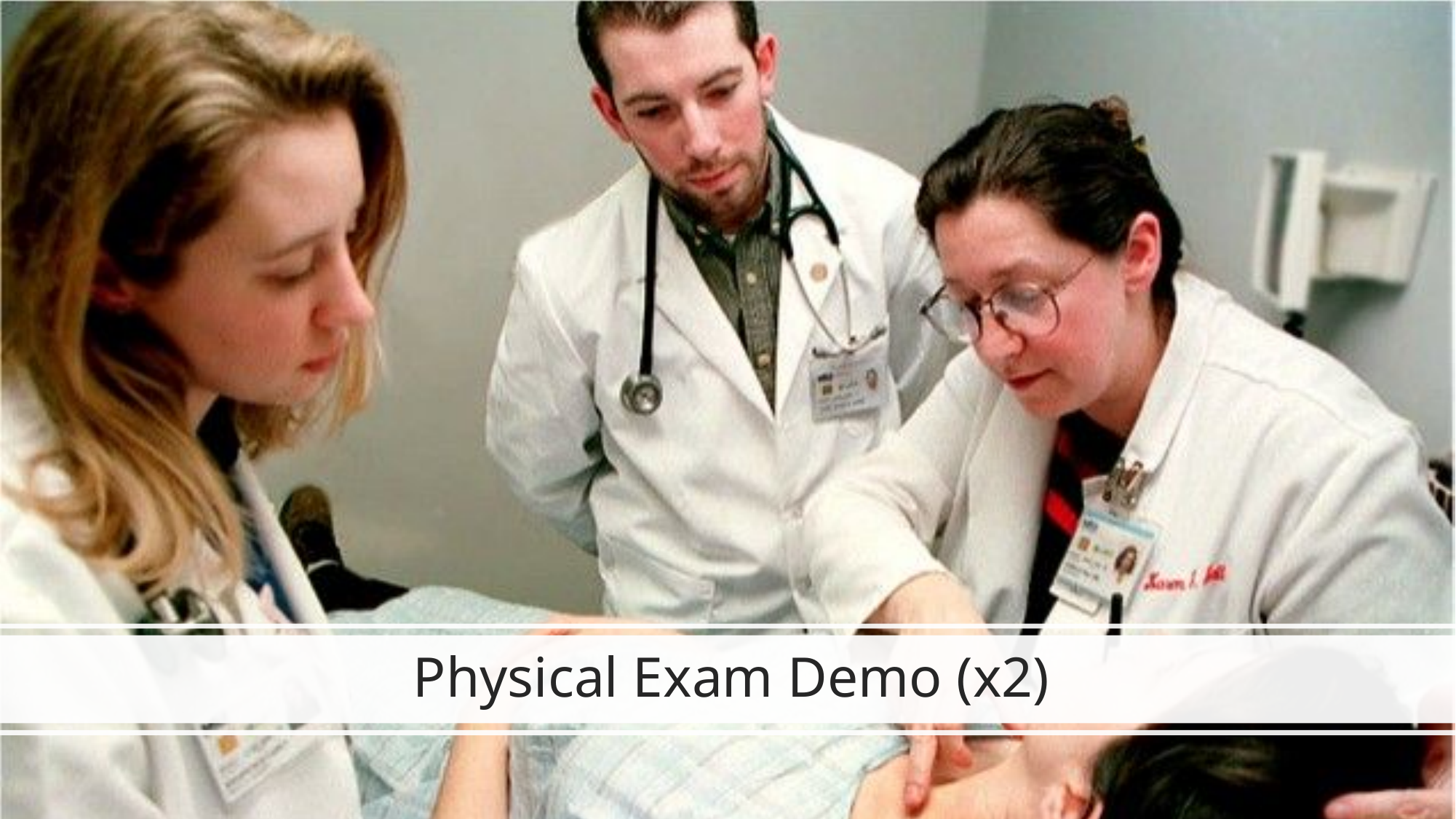

# Physical Exam Demo (x2)

## Slide 10
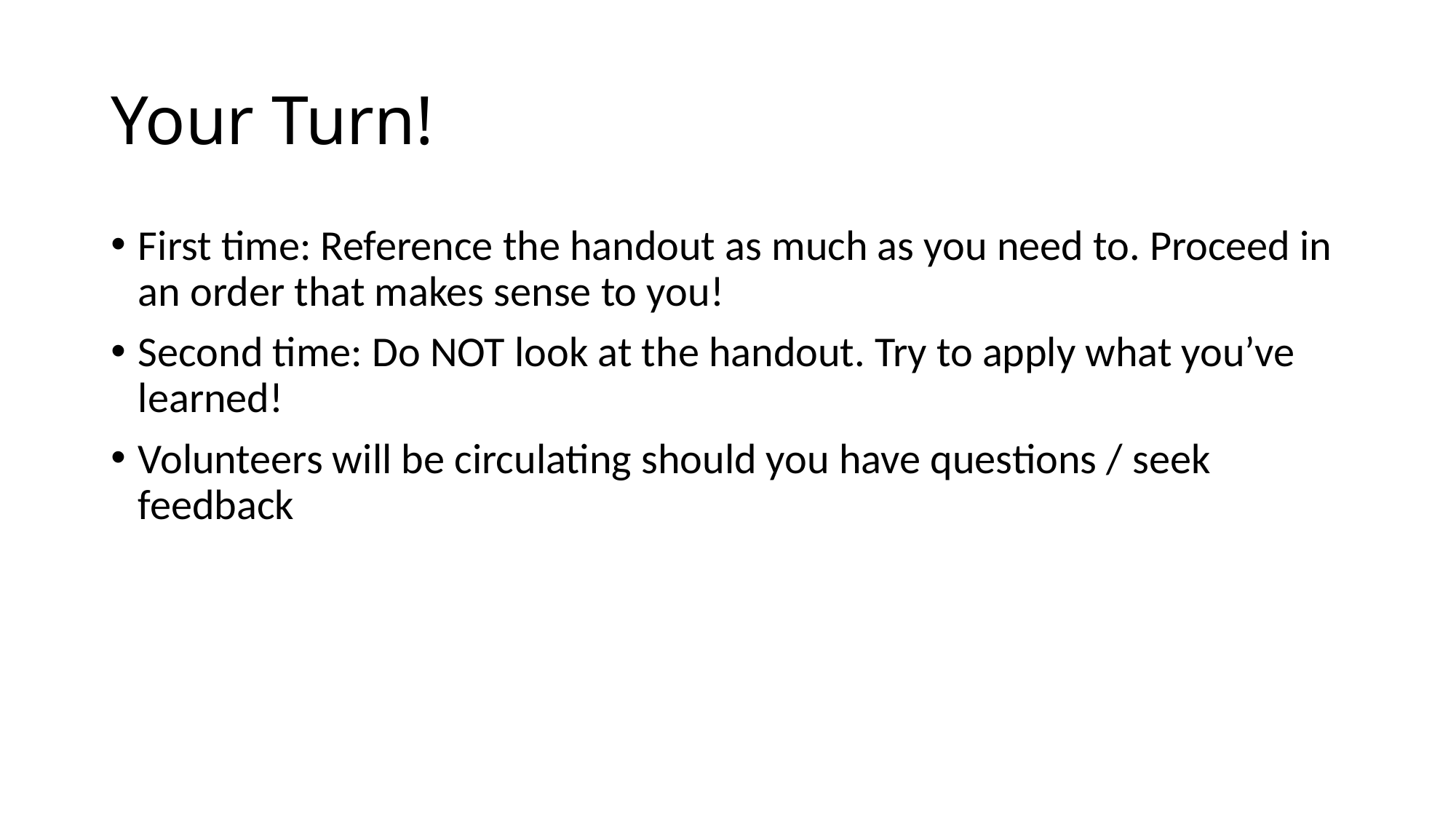

# Your Turn!
First time: Reference the handout as much as you need to. Proceed in an order that makes sense to you!
Second time: Do NOT look at the handout. Try to apply what you’ve learned!
Volunteers will be circulating should you have questions / seek feedback

## Slide 11
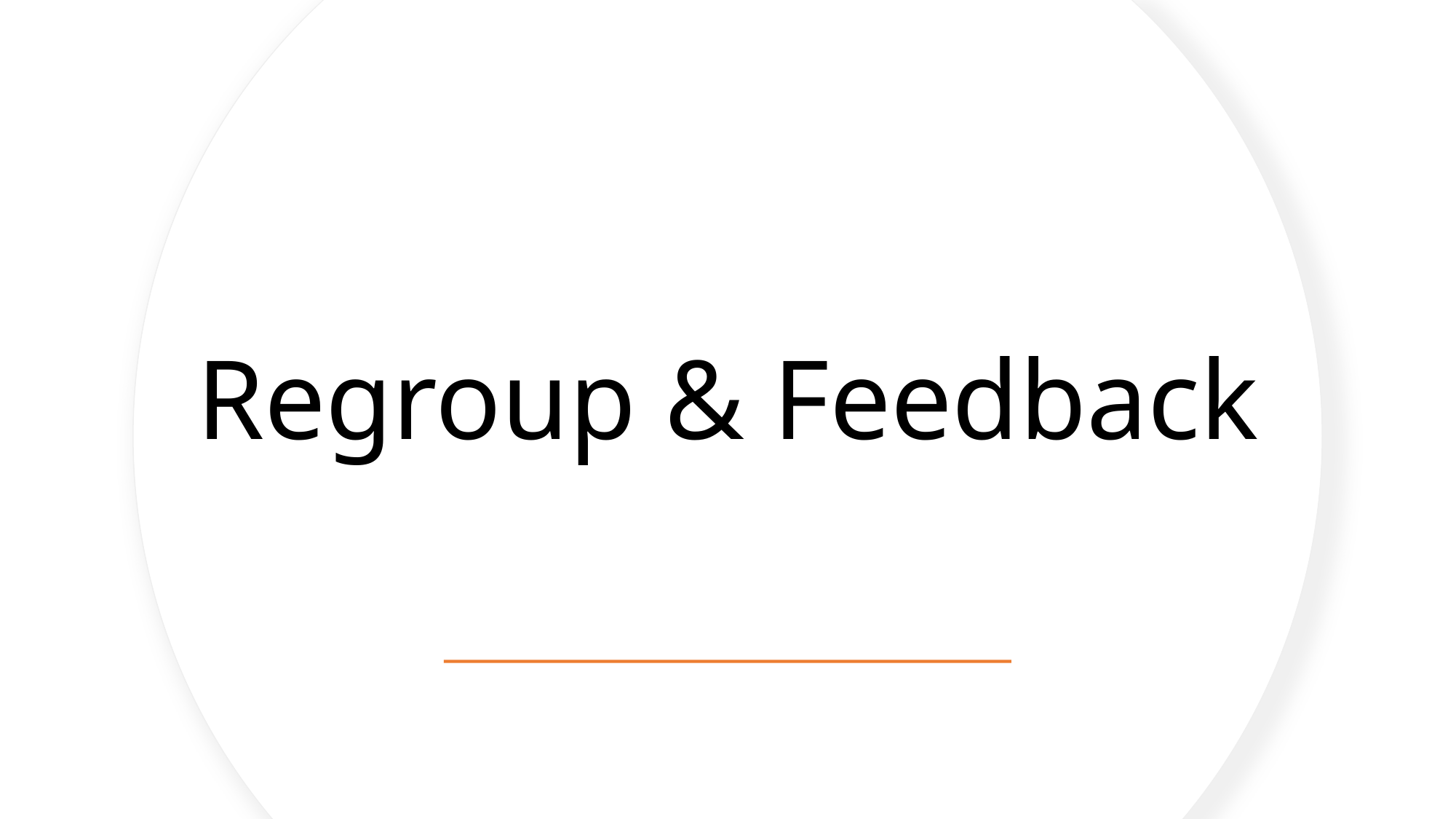

# Regroup & Feedback

## Slide 12
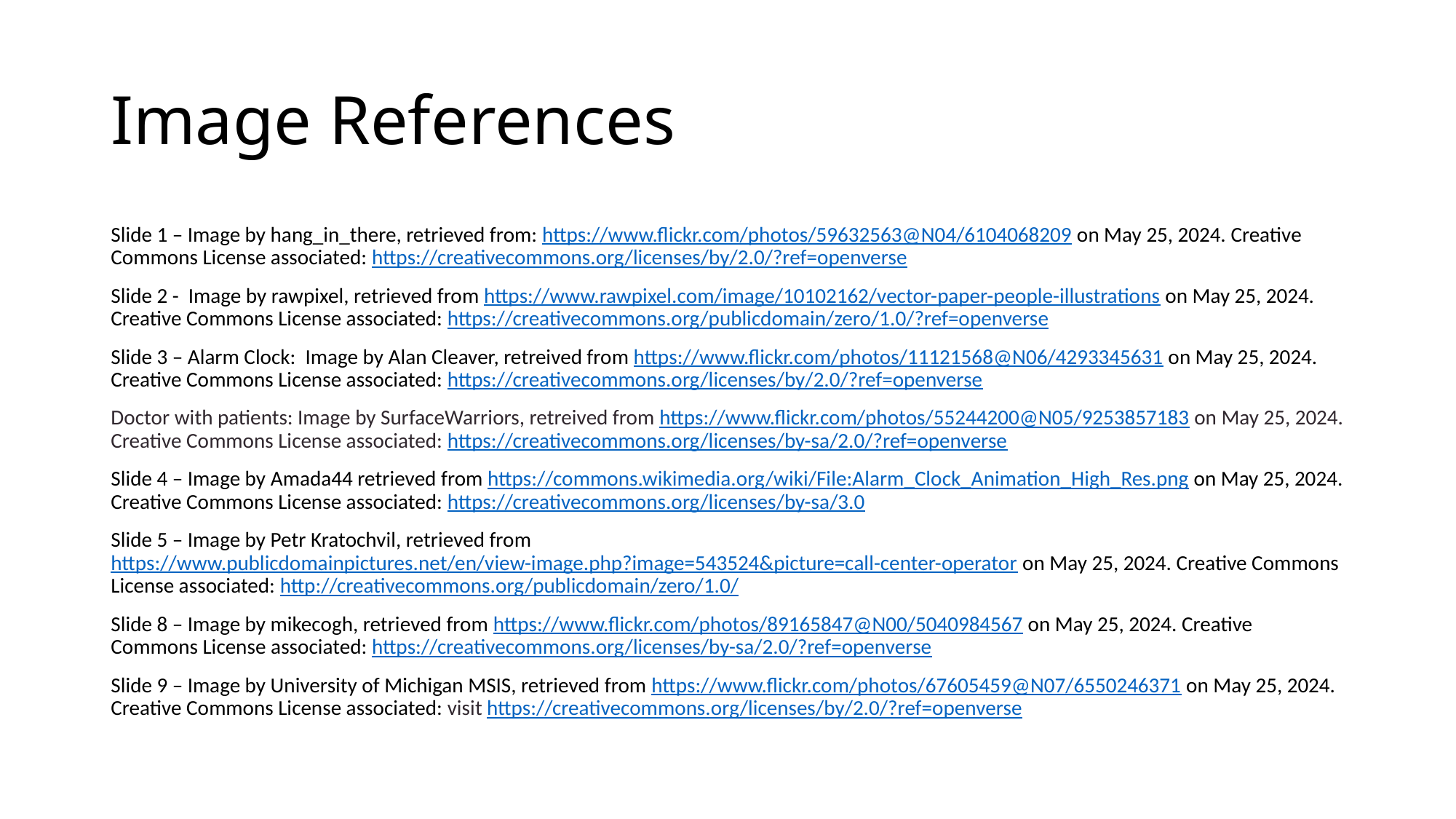

# Image References
Slide 1 – Image by hang_in_there, retrieved from: https://www.flickr.com/photos/59632563@N04/6104068209 on May 25, 2024. Creative Commons License associated: https://creativecommons.org/licenses/by/2.0/?ref=openverse
Slide 2 -  Image by rawpixel, retrieved from https://www.rawpixel.com/image/10102162/vector-paper-people-illustrations on May 25, 2024. Creative Commons License associated: https://creativecommons.org/publicdomain/zero/1.0/?ref=openverse
Slide 3 – Alarm Clock:  Image by Alan Cleaver, retreived from https://www.flickr.com/photos/11121568@N06/4293345631 on May 25, 2024. Creative Commons License associated: https://creativecommons.org/licenses/by/2.0/?ref=openverse
Doctor with patients: Image by SurfaceWarriors, retreived from https://www.flickr.com/photos/55244200@N05/9253857183 on May 25, 2024. Creative Commons License associated: https://creativecommons.org/licenses/by-sa/2.0/?ref=openverse
Slide 4 – Image by Amada44 retrieved from https://commons.wikimedia.org/wiki/File:Alarm_Clock_Animation_High_Res.png on May 25, 2024. Creative Commons License associated: https://creativecommons.org/licenses/by-sa/3.0
Slide 5 – Image by Petr Kratochvil, retrieved from  https://www.publicdomainpictures.net/en/view-image.php?image=543524&picture=call-center-operator on May 25, 2024. Creative Commons License associated: http://creativecommons.org/publicdomain/zero/1.0/
Slide 8 – Image by mikecogh, retrieved from https://www.flickr.com/photos/89165847@N00/5040984567 on May 25, 2024. Creative Commons License associated: https://creativecommons.org/licenses/by-sa/2.0/?ref=openverse
Slide 9 – Image by University of Michigan MSIS, retrieved from https://www.flickr.com/photos/67605459@N07/6550246371 on May 25, 2024. Creative Commons License associated: visit https://creativecommons.org/licenses/by/2.0/?ref=openverse
