# Supplementary material for: The Flipped Classroom Approach: A Feasible Way to Teach the Physical Exam in Spanish
Source: MedEdPORTAL. 2025 Jun 4;21:11532. doi: 10.15766/mep_2374-8265.11532 (PMC12134118; doi:10.15766/mep_2374-8265.11532)
Supplement: Supplementary file 1 — Introduction.mp4Vitals.mp4Cardiovascular.mp4Pulmonary.mp4Abdominal.mp4HEENT.mp4Neuro.mp4Workshop Student Handout.pptxWorkshop Slideshow.pptxSession Surveys.docx [file mep_2374-8265.11532-s001.zip › J. Session Surveys.docx]

**How comfortable do you feel doing the following with Spanish-speaking patients?**

|  | 1  Very Uncomfortable | 2  Somewhat Uncomfortable | 3  Neutral | 4  Somewhat Comfortable | 5  Very Comfortable |
| --- | --- | --- | --- | --- | --- |
| Introducing myself | ◯ | ◯ | ◯ | ◯ | ◯ |
| Building rapport | ◯ | ◯ | ◯ | ◯ | ◯ |
| Performing a thorough physical exam | ◯ | ◯ | ◯ | ◯ | ◯ |
| Performing an efficient physical exam | ◯ | ◯ | ◯ | ◯ | ◯ |
| Talking without an interpreter present | ◯ | ◯ | ◯ | ◯ | ◯ |
| Working collaboratively with an interpreter to do a physical exam | ◯ | ◯ | ◯ | ◯ | ◯ |

**To what degree do you agree with the following?**

|  | 1  Strongly Disagree | 2  Somewhat Disagree | 3  Neutral | 4  Somewhat Agree | 5  Strongly Agree |
| --- | --- | --- | --- | --- | --- |
| Learning Spanish is important for me | ◯ | ◯ | ◯ | ◯ | ◯ |
| Speaking Spanish with patients can improve their medical care | ◯ | ◯ | ◯ | ◯ | ◯ |
| More physicians in the U.S. should learn how to speak Spanish | ◯ | ◯ | ◯ | ◯ | ◯ |
| I would like to learn Spanish to better communicate with my patients | ◯ | ◯ | ◯ | ◯ | ◯ |

**Did you watch any of the Spanish physical exam module videos ahead of the session?**

◯ Yes, all of them ◯ Yes, some of them ◯ No

**If yes, how would you rate your satisfaction with the modules in the following areas?**

|  | 1  Very Unsatisfied | 2  Somewhat Unsatisfied | 3  Neutral | 4  Somewhat Satisfied | 5  Very Satisfied |
| --- | --- | --- | --- | --- | --- |
| Difficulty of Spanish phrases used | ◯ | ◯ | ◯ | ◯ | ◯ |
| Usefulness of phrases learned | ◯ | ◯ | ◯ | ◯ | ◯ |
| Quality of video | ◯ | ◯ | ◯ | ◯ | ◯ |
| Overall | ◯ | ◯ | ◯ | ◯ | ◯ |

**How comfortable do you feel doing the following with Spanish-speaking patients?**

|  | 1  Very Uncomfortable | 2  Somewhat Uncomfortable | 3  Neutral | 4  Somewhat Comfortable | 5  Very Comfortable |
| --- | --- | --- | --- | --- | --- |
| Introducing myself | ◯ | ◯ | ◯ | ◯ | ◯ |
| Building rapport | ◯ | ◯ | ◯ | ◯ | ◯ |
| Performing a thorough physical exam | ◯ | ◯ | ◯ | ◯ | ◯ |
| Performing an efficient physical exam | ◯ | ◯ | ◯ | ◯ | ◯ |
| Talking without an interpreter present | ◯ | ◯ | ◯ | ◯ | ◯ |
| Working collaboratively with an interpreter to do a physical exam | ◯ | ◯ | ◯ | ◯ | ◯ |

**How satisfied do you feel overall with the session?**

◯ Very unsatisfied ◯ Unsatisfied ◯ Neutral ◯ Satisfied ◯ Very Satisfied

**If you watched any of the videos ahead of time, how well did they prepare you for the workshop on a scale of 1 (not at all) to 5 (very well)?**

◯ 1 ◯ 2 ◯ 3 ◯ 4 ◯ 5 ◯ N/A

**What did the session do well (free response)?**

**What could be improved for next time / What else would you have liked to learn how to say in Spanish (free response)?**
